# Chilean Registry for Neuroendocrine Tumors: A Latin American Perspective

**DOI:** 10.1007/s12672-018-0354-5

**Published:** 2018-11-22

**Authors:** Mauricio P. Pinto, Matías Muñoz Medel, Diego Carrillo, Ignacio N. Retamal, M. Loreto Bravo, Yasna Valenzuela, Bruno Nervi, César Sánchez, Héctor Galindo, Carolina Ibañez, José Peña, Carlos Balmaceda, Jorge Madrid, Juan Briones, Javiera Torres, Flavia Nilo, Francisco J. Guarda, Juan Carlos Quintana, Pilar Orellana, Sebastián Mondaca, Francisco Acevedo, Daniel Vicentini, Miguel Cordova-Delgado, Gareth I. Owen, Marcelo Garrido

**Affiliations:** 1 Department of Hematology & Oncology, School of Medicine, Pontificia Universidad Católica de Chile, Diagonal Paraguay 362, 6th floor, Santiago, Chile; 2 Department of Internal Medicine, School of Medicine, Pontificia Universidad Católica de Chile, Santiago, Chile; 3 Health Technology Assesment unit, Pontificia Universidad Católica de Chile, Santiago, Chile; 4 Department of Pathology, School of Medicine, Pontificia Universidad Católica de Chile, Santiago, Chile; 5 Department of Endocrinology, School of Medicine, Pontificia Universidad Católica de Chile, Santiago, Chile; 6 Department of Nuclear Medicine, School of Medicine, Pontificia Universidad Católica de Chile, Santiago, Chile; 7 Department of Physiology, School of Biological Sciences, Pontificia Universidad Católica de Chile, Santiago, Chile

**Keywords:** Neuroendocrine tumors, Cancer registry, Chromogranin A, Overall survival

## Abstract

Neuroendocrine tumors (NETs) are relatively rare and highly heterogeneous neoplasms. Despite this, recent studies from North America and Central Europe have suggested an increase in incidence. In Latin America, NET data are scarce and scattered with only a few studies reporting registries. Our goal was to establish a NET registry in Chile. Here, we report the establishment and our first 166 NET patients. We observed a slight preponderance of males, a median age at diagnosis of 53 years and a median overall survival of 110 months. As anticipated, most tumors were gastroenteropancreatic (GEP). Survival analyses demonstrated that non-GEP or stage IV tumors presented significantly lower overall survival (OS). Similarly, patients with surgery classified as R0 had better OS compared to R1, R2, or no surgery. Furthermore, patients with elevated chromogranin A (CgA) or high Ki67 showed a trend to poorer OS; however, these differences did not reach statistical significance (log-rank test *p* = 0.07). To the best of our knowledge, this is the first report of a NET registry in Chile. Median OS in our registry (110 months) is in line with other registries from Argentina and Spain. Other variables including age at diagnosis and gender were similar to previous studies; however, our data indicate a high proportion of small-bowel NETs compared to other cohorts, reflecting the need for NET regional registries. Indeed, these registries may explain regional discrepancies in incidence and distribution, adding to our knowledge on this seemingly rare, highly heterogeneous disease.

**Electronic supplementary material:**

The online version of this article (10.1007/s12672-018-0354-5) contains supplementary material, which is available to authorized users.

## Introduction

Neuroendocrine tumors (NETs) are a group of highly heterogeneous neoplasms that arise from cells of the diffuse neuroendocrine system that extend throughout the body. Initially described over a century ago by Oberndorfer [1], NETs are characterized by the secretion of biologically active peptides or neuropeptides that give rise to a variety of clinical syndromes, including the carcinoid syndrome. Despite this, in certain cases, NETs can remain clinically silent and undiagnosed until advanced stage disease. Anatomically, NETs are frequently located in the gastrointestinal tract; however, they can also originate in other organs such as the pancreas, lungs, adrenal glands, and the thyroid. Indeed, the vast majority of NET cases (75%) originate in the gastroenteropancreatic (GEP) system [2] and these cases are collectively referred to as GEP-NETs.

Still regarded as relatively rare tumors, several studies have previously reported NET incidence rates across North American [3, 4], Western European [5–8], and Asian [9, 10] countries. Interestingly, studies in North America and the United Kingdom (UK) indicate a significant increase in the incidence of NETs over the last decades. Data from the Surveillance, Epidemiology, and End Results (SEER) program registry in the USA involved > 35,000 patients and compared the NET age-adjusted incidence over the 1973–2004 period. This study found a significant increase in NET incidence from 1.9 to 5.25 per 100,000 per year during this period [3]. Similarly, a study in Canada that involved > 5000 patients reported an increase in incidence from 2.48 to 5.86 per 100,000 per year over the period 1994–2009 [4]. Furthermore, a study in the UK that identified > 10,000 cases of gastrointestinal NETs during the 1971–2007 period also found an increase from 0.27 to 1.32 per 100,000 in men and from 0.35 to 1.33 per 100,000 in women [6]. Although this increase can be attributed to an improvement in diagnostic techniques and a rise in NET/cancer awareness, a definitive explanation for this phenomenon is still pending, as is the discrepancy in incidence between the UK and North America. These regional differences further highlight the need to establish and maintain regional and country-based tumor registries.

NET incidence and/or prevalence in Latin American countries remain largely unreported, and clinical literature is extremely scarce [11, 12]. An observational study in Argentina documented a total of 532 NET cases that included 461 GEP-NETs and 71 bronchial NETs [12]. This study demonstrated that 26.9% of GEP-NETs were located at the small bowel, followed by pancreas (25.2%), colon–rectum (12.4%), appendix (7.6%), gastric (6.9%), esophagus (2.8%), and duodenum (2.0%), with a further 16.3% reported as unknown origin. A NET registry from Brazil has documented baseline information on the first 1000 patients and classified the majority of tumors as thoracic (71.6%), followed by GEP-NETs (20.2%), head and neck (1.5%), skin (0.9%), genitourinary tract (0.6%), adrenal (0.4%), biliary (0.3%), prostate (0.3%), esophagus (0.1%), breast (0.2%), kidney (0.1%), ovary (0.2%), and 3.6% classified as unknown origin [11].These results again demonstrate the population-specific incidence of the NET classified malignancies.

Herein, we deliver the first multicentric Chilean registry of NET patients. Of the 166 patients currently incorporated into this registry, we report that 115 are GEP-NETs and 51 classified as non-GEP-NETs. Interestingly, in contrast to previously reported finding from other NET registries, the Chilean registry data shows a high proportion of small-bowel tumors (46%) and stage IV metastatic disease at diagnosis (62%). Patient characteristics (age, sex, etc.) and survival rates were similar to those reported in other regions.

## Materials and Methods

### The First Chilean NET Patient Registry: Participating Institutions

A team of specialists that included medical oncologists, endocrinologists, gastroenterologists, nurses, and molecular biologists generated this registry. The registry was funded by Novartis, Chile. Novartis had no access to patient raw data or any participation in the establishment of the database, data acquisition, or analysis. This study was designed as an observational, multicenter, prospective, and retrospective registry, and approved by the institutional review board and ethics committee in all participating institutions, in accordance with the Declaration of Helsinki, Good Clinical Practices, and Chilean regulations. Participating institutions with ethics approval included the following: Hospital Base de Valdivia, Hospital de Punta Arenas, Hospital Dr. Sotero del Rio, Hospital Base de Osorno, Hospital Regional de Concepcion, and Hospital Clinico de la Universidad de Chile. Written informed consent was obtained from all participating patients.

### Chromogranin A, Intraplatelet Serotonin, and 5-Hydroxy-Indoleacetic Acid

Chromogranin A (CgA) levels were obtained from plasma samples using an ELISA kit from EURODIAGNOSTICA. Similarly, intraplatelet (I-P) serotonin levels were obtained from plasma by high-performance liquid chromatography (HPLC). 5-Hydroxy-indoleacetic acid (5-HIAA) was also obtained by HPLC but using 24-h collected urine samples.

### Data Acquisition

The Chilean registry involved the development of an online database of NET patients. Collected patient data were entered into a virtual platform at www.clinicaldata.cl. Patients were enrolled starting in July 2015 through July 2017. Data were also retrospectively collected from medical records at participating institutions. The database consisted of 86 validated entries that included basic information, demographics, onset symptoms, tumor characteristics, diagnostic procedures, treatment regime (if any), and clinical outcome. All physicians received prior training and were responsible for entering data into the registry. To assess the quality of the data entered in www.clinicaldata.cl, a trained monitor for the study periodically visited each participating center to review the relevant patient medical records.

### Inclusion and Exclusion Criteria

The registry included adult individuals (> 18 years old), diagnosed with histologically confirmed NETs and at least 3 months of follow-up with access to clinical information. Exclusion criteria consisted of patients with missing or incomplete information, absence of clinical follow-up data, or those unable or unwilling to sign written informed consent.

### Statistical Analysis

Continuous variables entered in the registry were expressed as mean plus/minus standard deviation or by median values and range (minimum and maximum) values according to their distribution (normal vs. not normal). Categorical variables were expressed as percentages (%). Statistical comparisons among groups were performed by Student’s t test when data were normally distributed; otherwise, the Mann–Whitney *U* test was performed. The distribution of continuous variables for > 2 groups was analyzed by the ANOVA or Kruskal–Wallis test, depending on data normality. The differences in categorical variables were tested by Fisher’s exact test. Survival curves were calculated using the Kaplan–Meier estimate method and different variables were compared by the log-rank test. All statistical analyses were performed using GraphPad Prism 7 or R statistical software. All analyses were two-tailed and significance was set at *p* ≤ 0.05.

## Results

### Patient Population

This registry enrolled a total of 166 eligible NET patients. The median age at diagnosis was 53 years (range 23–85) and male patients accounted for 54.2% (*n* = 90) of the entries. The majority, 115 out of 166 tumors (64%) were registered as GEP-NETs in line with previous reports. Within GEP tumors, the most frequent primary tumor site was the small bowel (46%, *n* = 53). Tumors were predominantly metastatic at diagnosis, classified as stage IV (62%). The majority of patients also had an ECOG performance status 0–1 (92%) and their histology classified as low-grade, well-differentiated (56%). In 92 patients, Ki67 was assessed by immunohistochemistry with 43% being classified as intermediate grade/moderately differentiated with a Ki67 level in the 3–20% range. Basic information, clinico-pathological characteristics, and demographics of patients are summarized in Table 1.

**Table 1:** Demographic and biological characteristics of registered Chilean NET patients. * Thoracic NET cases includes broncho/pulmonary and thymic tumors; ** unknown site NETs include breast (not primary) and other with uncertain origin

	All patients	GEP	Pancreatic	Gastric	Small bowel	Colorectal	Appendicular	Hepatobiliary	Thoracic*	Unknown**
Median age/years (range)	53 (22–85)	52 (25–81)	48 (25–78)	58 (29–81)	55 (27–77)	52.5 (25–70)	42 (28–63)	60 (49–71)	46 (22–73)	61 (33–75)
Median OS/months	110	168	ND	31	168	83	ND	ND	ND	68
5-year OS rate (%)	71.5	78.5	75.1	ND	86.5	68.8	ND	ND	70	50
Gender *n* (%)	166 (100)	115 (100)	35 (100)	9 (100)	53 (100)	12 (100)	4 (100)	2 (100)	11 (100)	9 (100)
Male	90 (54)	64 (56)	21 (60)	4 (44.4)	32 (60)	5 (41.7)	1 (25)	1 (50)	7 (63.6)	3 (33.3)
Female	76 (46)	51 (44)	14 (40)	5 (55.6)	21 (40)	7 (58.3)	3 (75)	1 (50)	4 (36.4)	6 (66.7)
Tumor stage *n* (%)	145 (100)	102 (100)	30 (100)	7 (100)	51 (100)	9 (100)	3 (100)	2 (100)	11 (100)	8 (100)
Stage I	17 (12)	14 (14)	4 (13.3)	4 (57.1)	3 (5.9)	1 (11.1)	2 (66.7)	0 (0)	3 (27.3)	0
Stage II	17 (12)	8 (8)	4 (13.3)	0	3 (5.9)	0	1 (33.3)	0 (0)	2 (18.2)	3 (37.5)
Stage III	21 (14)	16 (15)	5 (16.7)	0	8 (15.7)	2 (22.2)	0	1 (50)	1 (9.1)	0
Stage IV	90 (62)	64 (63)	17 (56.7)	3 (42.9)	37 (72.5)	6 (66.7)	0	1 (50)	5 (45.5)	5 (62.5)
ECOG *n* (%)	129 (100)	82 (100)	25 (100)	6 (100)	40 (100)	7 (100)	3 (100)	2 (100)	10 (100)	8 (100)
0	49 (38)	32 (39)	10 (40)	2 (33.3)	16 (40)	1 (14.3)	3 (100)	1 (50)	4 (40)	1 (12.5)
1	69 (54)	43 (52)	13 (52)	3 (50)	20 (50)	6 (85.7)	0	1 (50)	6 (60)	6 (75)
2	7 (5)	6 (8)	2 (8)	1 (16.7)	3 (7.5)	0	0	0	0	1 (12.5)
3	4 (3)	1 (1)	0	0	1 (2.5)	0	0	0	0	0
Tumor grade *n* (%)	159 (100)	111 (100)	34 (100)	8 (100)	51 (100)	12 (100)	4 (100)	2 (100)	10 (100)	8 (100)
1	90 (56)	67 (60)	21 (61.8)	2 (25)	36 (70.6)	6 (50)	2 (50)	0	5 (50)	2 (25)
2	34 (22)	31 (28)	10 (29.4)	3 (37.5)	14 (27.4)	4 (33.3)	0	0	0	0
3	35 (22)	13 (12)	3 (8.8)	3 (37.5)	1 (2)	2 (16.7)	2 (50)	2 (100)	5 (50)	6 (75)
Ki-67 *n* (%)	92 (100)	71 (100)	19 (100)	8 (100)	29 (100)	9 (100)	4 (100)	2 (100)	8 (100)	4 (100)
0–2%	27 (29)	25 (35)	6 (31.6)	4 (50)	13 (44.8)	3 (33.3)	3 (75)	0	2 (25)	0
3–20%	39 (43)	33 (47)	9 (47.4)	4 (50)	15 (51.7)	5 (55.6)	0	0	2 (25)	1 (25)
> 20%	26 (28)	13 (18)	4 (21)	0	1 (3.5)	1 (1.1)	1 (25)	2 (100)	4 (50)	3 (75)
CgA *n* (%)	57 (100)	52 (100)	21 (100)	3 (100)	23 (100)	3 (100)	0	2 (100)	3 (100)	1 (100)
< 100 ng/mL	27 (47)	23 (44)	8 (38.1)	1 (33.3)	12 (52.2)	2 (66.7)	0	0	2 (66.7)	1 (100)
> 100 ng/mL	30 (53)	29 (56)	13 (61.9)	2 (66.7)	11 (47.8)	1 (33.3)	0	2 (100)	1 (33.3)	0
I-P serotonin *n* (%)	45 (100)	43 (100)	8 (100)	1 (100)	30 (100)	4 (100)	0	0	0	0
400–800 ng/10^9^ plat.	12 (27)	10 (23)	2 (25)	0	6 (20)	2 (50)	0	0	0	0
> 800 ng/10^9^ plat.	33 (73)	33 (77)	6 (75)	1 (100)	24 (80)	2 (50)	0	0	0	0
5-HIAA *n* (%)	52 (100)	49 (100)	11 (100)	0	33 (100)	4 (100)	0	0	0	0
< 100 μmol/h	21 (40)	18 (37)	7 (63.6)	0	8 (24.2)	2 (50)	0	0	0	0
> 100 μmol/h	31 (60)	31 (63)	4 (36.4)	0	25 (75.8)	2 (50)	0	0	0	0

### NET Biomarkers and Quality of Surgery

Chromogranin A (CgA), intraplatelet (I-P) serotonin, and 5-hydroxy-indoleacetic acid (5-HIAA) were measured as diagnostic biomarkers in subsets of patients. First, CgA levels from 57 patients were obtained from plasma samples using an ELISA kit. In the subset of GEP-NETs, I-P serotonin was measured in 45, and 5-HIAA in 52 patients respectively. Table 1 demonstrates that biomarkers were elevated in the majority of patients. CgA levels were elevated (> 100 ng/ml) in 53%. Similarly, I-P serotonin and 5-HIAA levels were increased (> 800 ng/10^9^ platelets and > 100 μmol/h, respectively) in 73% and 60% of cases, respectively. Finally, patient surgery was categorized as R0 when no macroscopic disease was visible post-surgery; in our cohort 124 out of 166 patients had surgery (74.6%); within this group, 52% were classified as R0.

### Survival Rates

**Fig. 1:**
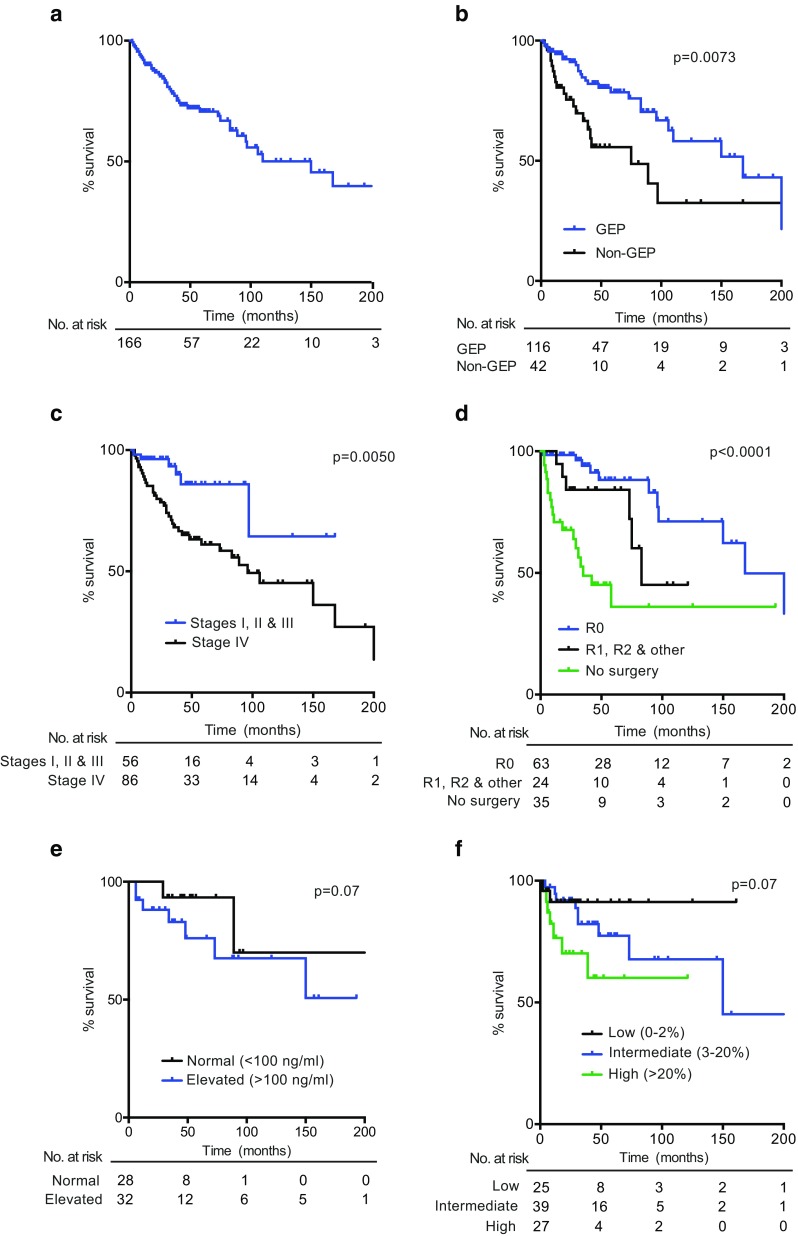
Kaplan–Meier survival curves in Chilean NET patients. **a** For all patients recorded in this registry (*n* = 166). **b** According to tumor primary site, comparing GEP versus non-GEP (Log Rank test *p* = 0.0073). **c** By tumor stage, comparing stage I, II and III against stage IV (log-rank test *p* = 0.0050). **d** By quality of surgery, comparing no surgery, R0, or R1+R2+other grouped (log-rank test *p* < 0.0001). **e** By CgA levels comparing normal (< 100 ng/ml) versus elevated (≥ 100 ng/ml) (log-rank test *p* = 0.07). **f** By Ki67, comparing low (0–2%), intermediate (3–20%), and high (> 20%) levels (log-rank *p* = 0.07). The number of patients at risk over time, in each case, is indicated at the bottom of every graph

Median overall survival (OS) for all registered patients was 110 months with a 5-year survival rate of 71% (see Table 1 and Fig. 1a). When analyzed by tumor primary site, the most favorable prognosis was observed in the small bowel (median OS 168 months, 5-year survival 86.5%) and the least among gastric tumors with a median OS of 31 months. Figure 1 shows Kaplan–Meier estimate curves: for all patients (Fig. 1a), by tumor primary site (GEP versus non-GEP; Fig. 1b, log rank test *p* = 0.0073), by cancer stage (Fig. 1c, log rank test *p* = 0.0050), by quality of surgery (Fig. 1d, log rank test *p* < 0.0001), by serum CgA concentration (Fig. 1e) and by Ki67 index (Fig. 1f). Briefly, patients with GEP tumors had significantly better OS rates than non-GEP (Fig.1b). As expected, OS rates were lower in stage IV patients than with stage I, II, and III combined (Fig. 1c). Patients with optimal surgery classified as R0 had significantly better OS (Fig. 1d) and patients with elevated CgA levels or high Ki67 had worse OS (Fig. 1e, f); however, these differences did not reach statistical significance (*p* = 0.07). The number of patients at risk over time, in every case, is indicated at the bottom of every graph.

### NET Primary Site Distribution in Chilean in Comparison to Other Registries

Table 2 shows the distribution by primary site across various NET registries. First, Table 2A compares primary site distribution only in GEP-NET cohorts in China [13], Argentina [12], and Chile. Second, Table 2B compares primary site distribution excluding pancreas (GI-NET) in cohorts from England [6], Japan [14], and Chile. Then, Table 2C compares the distribution of primary sites among cohorts that include all NETs from the USA (the SEER database) [3], Spain [5], Norway [15], and Chile. The Brazilian NET registry reported tumor sites as thoracic was not included in Table 2.

**Table 2:** Comparative table of Chilean data compared to published NET registries. Distribution by tumor primary site

	A. GEP-NETs cohorts: primary site proportions			B. GI-NETs cohorts: primary site proportions			C. All NETs cohorts: primary site proportions			
Primary site	China	Argentina	Chile	England	Japan	Chile	USA	Spain	Norway	Chile
Stomach	27%	7%	8%	12%	15%	11%	5%	6%	3%	5%
Small bowel	6%	29%	46%	29%	19%	65%	23%	16%	12%	32%
Colon	3%	12%	10%	13%	2%	16%	4%	5%	4%	7%
Appendix	2%	#	4%	38%	NR	3%	3%	9%	4%	2%
Rectum	30%	#	#	8%	55%	#	15%	6%	2%	#
Pancreas	31%	25%	30%	–	–	–	7%	34%	3%	21%

*#* Indicates these were recorded as colon, *NR* no reported value

## Discussion

To the best of our knowledge, this is the first NET registry in the Pacific South American region that describes epidemiologic and clinico-pathologic characteristics of patients. To date, the Chilean registry contains a relatively low number of recorded cases (*n* = 166); however, it is important to note that Chile has a population of approximately 17.5 million which is notably lower than other countries with NET registries [16]. While the largest NET database is currently maintained by the SEER program in the USA [3], there exist only two reports of NET registries in Latin America [11, 12], and from these, only an observational study from Argentina reports on 532 NET cases which include relevant clinical data.

Overall, the Chilean registry confirms that NETs are a highly heterogeneous group of neoplasms with a wide variety of clinical presentations and outcomes. NETs are commonly considered indolent tumors, especially when compared to carcinomas; however, the prognosis among Chilean patients was highly variable with median OS values that ranged from 168 months in small-bowel tumors, down to 31 months in gastric tumors. Despite this, the median OS for our study was 110 months, which is in line with Spanish and Argentinean NET registries that reported 144 and 121 months OS, respectively [5, 12], and possibly reflecting the recent common heritage between these nations. Likewise, the median age at diagnosis in Chilean patients was 53 years, a value comparable to registries in China (53 years) [13] and Argentina (53.2 years) [12]. The Chilean registry also demonstrated a slight preponderance of males over females, a trend similar to that reported in a Spanish national NET registry [5], yet differing from other reports that show a higher NET prevalence among the female population [3, 17].

Chromogranin A (CgA) is widely used as a biomarker for NET diagnosis and monitoring [18–20]. Although there are several methods to measure CgA levels, the only clinically validated method available in Chile is by ELISA; for consistency, all CgA measurements in our registry were performed at the same center using this method. Also, general consensus establishes ~ 100 ng/ml as a cutoff for normal CgA levels. However, as occurs with NET prevalence and incidence, the normal range of CgA levels in Latin American patients is somewhat uncertain. Currently, our research group is working on the assessment of CgA levels in Chilean healthy subjects and NET patients in order to obtain a more accurate CgA cutoff value.

In relation to the primary tumor sites, we found that the majority of recorded tumors were reported as GEP-NETs, in accordance with previous registries [5, 17]. However, when analyzed in further detail, the percentages of this classification of tumor displayed discrepancies across different geographic areas, for example, the percentage of small-bowel tumors in our cohort was 46%, a value higher than the 29% reported in Argentina [12] and strikingly different from the 6% in a Chinese registry [13]. Conversely, the proportion of stomach (27%) and rectum (30%) tumors in the Chinese registry was notoriously higher than that reported in the present study (8% and < 10% respectively, see comparative Table 2A). The high proportion of small-bowel NETs in our population study was even more evident when we compared other registries reporting only GI-NETs from England [6] and Japan [14] (see comparative Table 2B). Furthermore, comparative Table 2C shows that the proportion of small-bowel tumors in our cohort (32%) is twofold the reported value in the Spanish registry (16%) and higher than the SEER database (23%). Undoubtedly, a definitive explanation for this phenomenon remains pending; however, we speculate this is likely derived from a combination of ethnic and environmental influences, as genetically the Chilean population possess a high European ancestry component combined with Native American [21] that might explain some similarities with the Spanish registry [5] and the remarkable differences with Asian registries [13, 14]. The comparative GEP-NET data alone (Table 2A) illustrates the need for national and regional databases. Regarding origin, Argentina and Chile in South America tend to have a lower percentage of stomach tumors and a higher proportion of small bowel; notably, an inverse distribution is observed in the available data from China. Furthermore, the combined proportion of GEP-NET/colon (this includes colon, appendix, and rectum) in Argentina (12%) and Chile (10%) are quite similar, in sharp contrast with the GEP-NET/colon percentage in China (30%). Apart from ethnic differences, an alternative explanation for these regional discrepancies is that several members of the medical team that elaborated this Chilean registry are gastroenterologist surgeons, and therefore, the high percentage of small-bowel tumors could be overestimated due to a registration bias. Similarly, we noticed a relatively small percentage of thoracic NETs in our registry (6.6%) that includes bronchial (*n* = 8) and thymus (*n* = 3) tumors. The SEER database indicates up to 23% of thoracic NETs among Caucasian males [3]. Our cancer center is a national reference center for breast and gastric cancer patients, and therefore, the lack of thoracic NETs could also be attributed to a registration bias. Prospectively, our registry will seek to incorporate more centers in order to avoid this ascertainment bias.

Another interesting finding of our study is the significant proportion of stage IV metastatic patients (62%, Table 1); this is also notoriously different from other registries including the SEER database (21%) or the national registry from Spain (44%). Several factors could potentially explain these numbers: firstly, the Chilean registry is led by oncologists that frequently deal with more advanced disease patients compared to other medical professionals in the area such as endocrinologists or gastroenterologists, and as mentioned above, this could be another registration bias in our study. Second, this could be attributed to the late diagnosis of patients; in general, NETs are difficult to diagnose; as explained above, these tumors can sometimes remain asymptomatic for several years until the onset of metastatic disease. Finally, this could be attributed to a socioeconomic factor in Chile, the relatively high costs of treatment and diagnostic tests along with lack of medical insurance coverage may result in many patients receiving diagnosis and medical attention at a more advanced stage.

In summary, our study presents for the first time a Chilean NET registry including a wide range of primary sites, confirming the previously reported high heterogeneity of NETs. As discussed above, reported demographics and basic clinico-pathological characteristics of patients are in line with other NET registries. Remarkably, our study shows an unusual high proportion of small-bowel NETs and advanced stage IV tumors. The regional differences in NET primary site proportions and sub-types highlight the need for country-based NET databases in order to identify population-specific bias and may provide the basis for a better understanding of the regional discrepancies in incidence and distribution. A definitive explanation for these discrepancies remains pending and further registry-guided investigation will add to the knowledge on this seemingly rare and highly heterogeneous disease.

## Electronic supplementary material

ESM 1 (PNG 285 kb)

High resolution image (TIF 798 kb)

ESM 2 (AI 1467 kb)
