# Supplementary figures and images for: Chilean Registry for Neuroendocrine Tumors: A Latin American Perspective
Source: Horm Cancer. 2018 Nov 22;10(1):3–10. doi: 10.1007/s12672-018-0354-5 (PMC6334732; doi:10.1007/s12672-018-0354-5)

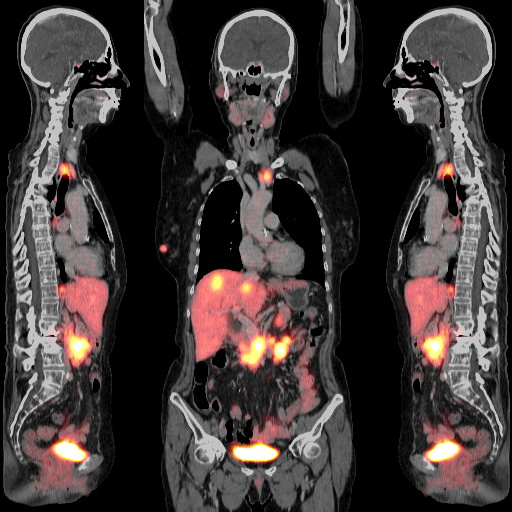

Supplement: Supplementary file 1 — (PNG 285 kb) [file 12672_2018_354_Fig2_ESM.png]

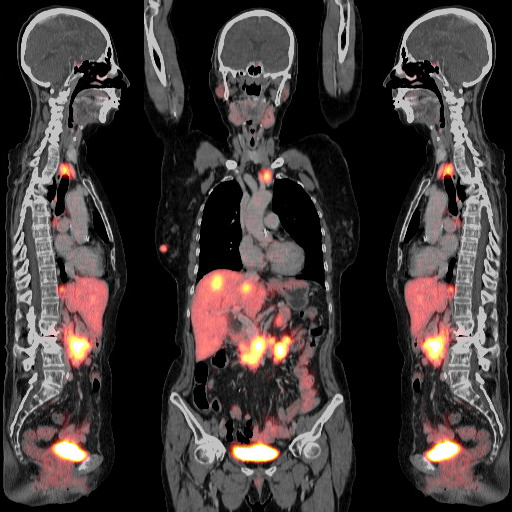

Supplement: Supplementary file 2 — High resolution image (TIF 798 kb) [file 12672_2018_354_MOESM1_ESM.tif]
